# Metabolic Response of Pancreatic Carcinoma Cells under Treatment with Dichloroacetate

**DOI:** 10.3390/metabo11060350

**Published:** 2021-05-30

**Authors:** Benedikt Feuerecker, Philipp Biechl, Christian Veltkamp, Dieter Saur, Wolfgang Eisenreich

**Affiliations:** 1 Department of Nuclear Medicine, School of Medicine, Technische Universität München, 81675 Munich, Germany; 2 German Cancer Consortium (DKTK), Partner Site München, and German Cancer Research Center (DKFZ), 69120 Heidelberg, Germany; 3 Department of Radiology, School of Medicine, Technische Universität München, 81675 Munich, Germany; 4 Bavarian NMR Center—Structural Membrane Biochemistry, Department of Chemistry, Technische Universität München, 85748 Garching, Germany; philipp.biechl@riseup.net; 5 Department of Internal Medicine II, School of Medicine, Technische Universität München, 81675 Munich, Germany; chrveltkamp@googlemail.com (C.V.); dieter.saur@tum.de (D.S.)

**Keywords:** dichloroacetate (DCA), isotopologue profiling, pancreatic cancer, [U-^13^C_6_]glucose

## Abstract

In modern oncology, the analysis and evaluation of treatment response are still challenging. Hence, we used a ^13^C-guided approach to study the impacts of the small molecule dichloroacetate (DCA) upon the metabolic response of pancreatic cancer cells. Two different oncogenic PI3K-driven pancreatic cancer cell lines, 9580 and 10,158, respectively, were treated with 75 mM DCA for 18 h. In the presence of [U-^13^C_6_]glucose, the effects of DCA treatment in the core carbon metabolism were analyzed in these cells using gas chromatography–mass spectrometry (GC/MS). ^13^C-enrichments and isotopologue profiles of key amino acids revealed considerable effects of the DCA treatment upon glucose metabolism. The DCA treatment of the two pancreatic cell lines resulted in a significantly decreased incorporation of [U-^13^C_6_]glucose into the amino acids alanine, aspartate, glutamate, glycine, proline and serine in treated, but not in untreated, cancer cells. For both cell lines, the data indicated some activation of pyruvate dehydrogenase with increased carbon flux via the TCA cycle, but also massive inhibition of glycolytic flux and amino acid biosynthesis presumably by inhibition of the PI3K/Akt/mTORC axis. Together, it appears worthwhile to study the early treatment response in DCA-guided or accompanied cancer therapy in more detail, since it could open new avenues for improved diagnosis and therapeutic protocols of cancer.

## 1. Introduction

Pancreatic cancer is one of the most malignant diseases. Unfortunately, 5-year survival rates remain below 5% [1]. The predominant form of pancreatic cancer is pancreatic ductal adenocarcinoma (PDAC) [2,3,4]. PDAC has been reported to develop via acinar-ductal metaplasia (ADM) and precursor lesions such as pancreatic intraepithelial neoplasia (PanIN), mucinous cystic neoplasia, atypical flat lesions (AFLs) and intraductal papillary mucinous neoplasia (IPMN) [2,3,4]. In >90% of PDAC, mutationally activated KRAS is present and represents at the same time the most frequent (>90%) and earliest genetic alteration [2,4,5]. Various studies have contributed to highlighting the importance of Kras signaling in the cascade of tumor development, growth and maintenance of metastatic lesions [2,6,7]. As KRAS oncoprotein activity drives PDAC maintenance in a large subset of PDAC patients, KRAS seemed to be a desirable target for direct inhibition. Nevertheless, all clinical attempts so far to drug this protein have failed, resulting in the assumption that KRAS is undruggable [8]. As a next step, new approaches focus on the identification of crucial yet druggable downstream effectors of oncogenic KRAS. Among the pathways that contribute to PDAC initiation, maintenance and tumor progression, various downstream effectors are affected, such as Raf/Mek/Erk, phosphatidylinositol 3-kinase (PI3K)/3-phosphoinositide-dependent protein kinase-1 (Pdk1)/Akt, RalGDS/p38MAPK, Rho and Rac, p120GAP [2,9,10]. While all of these pathways take part in oncogenesis, it is thought that KRAS is mainly passed through three major pathways, including Raf/Mek/Erk, phosphatidylinositol 3-kinase (PI3K)/3-phosphoinositide-dependent protein kinase-1 (Pdk1)/Akt and the Ral guanine nucleotide exchange factor pathway [2]. The phosphatidylinositol 3-kinase (PI3K)/3-phosphoinositide-dependent protein kinase-1 (Pdk1)/Akt is among the pathways that are a promising target. Frequently, genetic mutations in the phosphatidylinositol-3 kinase (PI3K) and the mitogen-activated protein kinase (MAPK) pathways are implicated in pancreatic cancer [11]. Clinical studies targeting this signaling cascade, unfortunately, were not successful so far [11].

In an aim to use a metabolism-oriented approach to target selected pancreatic cancer cells, we used the orphan drug dichloroacetate (DCA). DCA is a small molecule that was proposed as a metabolic modulator [12] that inherits the potential to overcome chemoresistance [13]. DCA was evaluated in the treatment of cancer alone or in combination with different additional agents such as metformin [14] or arginase in triple-negative breast cancer cells [15] as well in several experimental investigations [16,17]. This small molecule of 150 Da has high bioavailability [18], and it was shown in vitro that the compound activates pyruvate dehydrogenase (PDH) by inhibition of pyruvate decarboxylase-kinase (PDK), which is the inhibitor of PDH [19]. Characterization of pharmacokinetics and metabolism of DCA was analyzed in several earlier studies. With Sprague Dawley rats, the pharmacokinetics and metabolism of DCA were monitored with [^14^C]-enriched and [^13^C]-labelled DCA [20]. It was demonstrated that CO_2_, glycine, and oxalate were major products of DCA metabolism, and one dose of DCA changed the elimination of subsequent doses [20]. In human participants, following the administration of [1,2-^13^C_2_]DCA to healthy volunteers, the kinetics of DCA metabolization into monochloroacetate (MCA), glyoxylate, glycolate and oxalate were determined in human plasma using GS-MS [21]. Further, it was shown that through biotransformation to dichloroacetate, chloral hydrate inhibits maleylacetoacetate isomerase and tyrosine catabolism in humans [22].

We hypothesized that DCA might inherit the potential to affect metabolism in two PI3K-mutated pancreatic cancer cell lines. In order to further explore this, we have exploited a previously used method to monitor cancer metabolism by labeling experiments starting from ^13^C-labeled glucose as a tracer [23,24]. Indeed, the resulting labeling profiles in metabolic products can glean downstream metabolic pathways and fluxes, as shown for MCF7 breast cancer cells cultivated under various conditions [23,25]. Using similar methods, it was demonstrated that a metabolism-oriented approach helps to identify metabolic heterogeneity in lung cancers, which demonstrated higher lactate metabolism in cancer but not in benign cells [26,27,28], and to detect effects of alpha-radiation in malignant cancer cells [24]. The complex interplay between different metabolic pathways and metastatic/oncological behavior can be further investigated using this rather untargeted approach.

It was the purpose of this study to use the ^13^C-labelling approach followed by the GC-MS analysis of protein-derived amino acids in order to elucidate the effects of DCA upon the core metabolic network of two selected pancreatic cancer cell lines. For both cancer cell lines, we could demonstrate that DCA treatment results in effective reduction in ^13^C-flux from exogenous glucose into amino acids derived from glycolysis as well as from TCA cycle intermediates. On this basis, DCA seems to affect the PI3K/mTORC axis rather than the PDK/PDH enzymes alone.

## 2. Results

### 2.1. Proliferation/Viability

**Figure 1:**
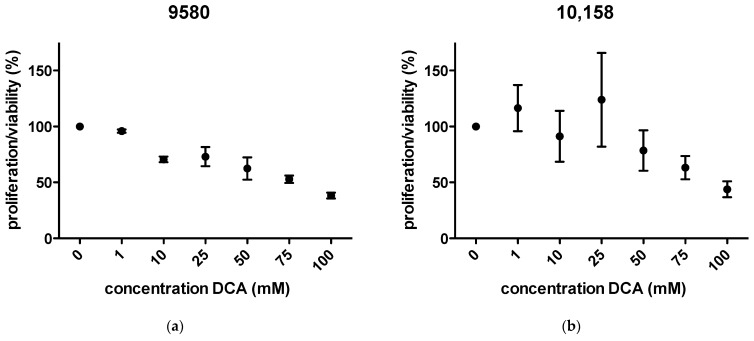
WST-1 viability/proliferation assays under treatment with DCA. Significant decrease in viability/proliferation in 9580 (**a**) and 10,158 cells (**b**) is shown at concentrations of 75 mM.

Proliferation/viability was decreased in a dose-dependent pattern in 9580 cells after incubation with DCA for 24 h. In 10,158 cells, proliferation showed a slight increase at concentrations up to 25 mM. Higher concentrations of DCA showed a decrease in viability. The approximate IC_50_ was at 75 mM in both cell lines (Figure 1a,b). In the ^13^C-labelling experiments, the cells were exposed to 75 mM DCA.

### 2.2. Isotopologue Distribution and ^13^C-Enrichment in Selected Amino Acids

**Figure 2:**
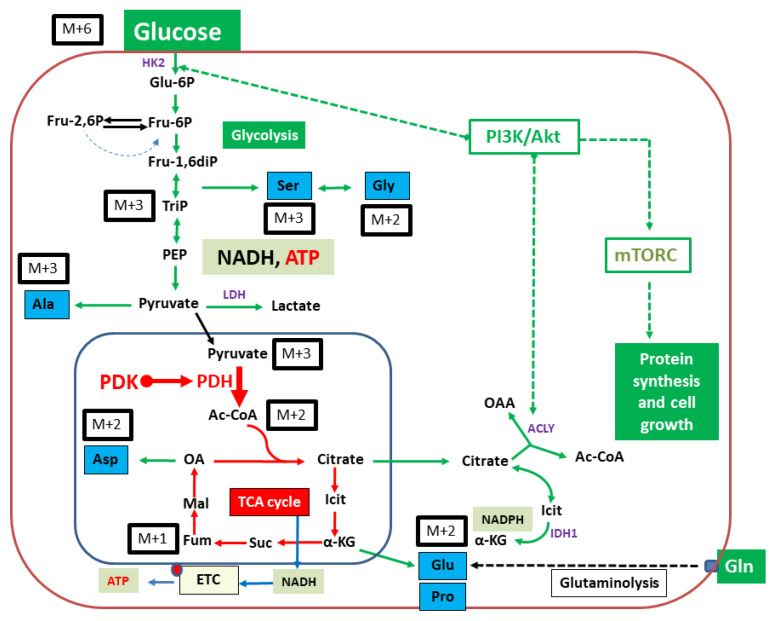
Typical core metabolism in cancer cells controlled by upregulated PI3K/Akt/mTORC. Green arrows indicate metabolic flux at high efficiencies. Notably, glucose uptake and utilization via glycolysis are upregulated. Flux via the TCA cycle and oxidative phosphorylation is downregulated by PDH inactivation due to phosphorylation catalyzed by PDK. When starting from [U-^13^C_6_]glucose (M + 6) as a carbon substrate, the resulting ^13^C-isotopologues in key metabolites and amino acids (in blue boxes) are indicated. M + 3 and M + 2 indicate molecules carrying three or two ^13^C-atoms, respectively. PDH, pyruvate dehydrogenase; PDK, pyruvate dehydrogenase kinase, ETC, electron transfer chain; HK2, hexokinase 2; ACLY, ATP-citrate lyase; IDH1, isocitrate dehydrogenase 1.

As examples for oncogenic PI3K-driven pancreatic cancer cells, we have selected PI3K mutated 9580 and 10,158 cell lines that were previously derived from pancreatic cancer isolates in mice bearing orthotopic pancreatic cancer. These cell lines were incubated for 18 h in a DMEM medium containing 75 mM DCA and 5 mM [U-^13^C_6_]glucose. After this period, the cells were harvested. Cells were hydrolyzed under acidic conditions. The resulting amino acids alanine, aspartate, glutamate as well as glycine, proline and serine were silylated. Analysis was performed with GC-MS. By careful evaluation of the mass patterns, the relative abundances of molecules carrying a given number of ^13^C-atoms were determined. This resulted in the isotopologue patterns. Additionally, the below reported overall ^13^C-contents in these amino acids were acquired. Derived amino acids from glycolytic intermediates, such as 3-phosphoglycerate (glycine, serine) and pyruvate (alanine) as well as intermediates from the TCA cycle, such as oxaloacetate (aspartate), or alpha-ketoglutarate (glutamate and proline) followed well established human metabolic pathways (please see Figure 2). Keeping this observation in mind, DCA treatment-related effects on glucose flux (e.g., glycolysis) and further downstream carbon metabolism (e.g., via PDH and the TCA cycle) should become transparent.

### 2.3. Overall ^13^C-Contents of Amino Acids

**Figure 3:**
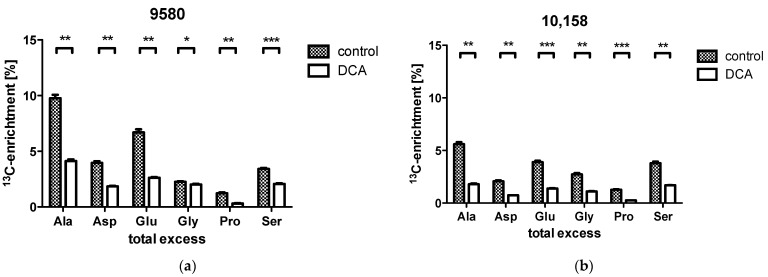
Overall ^13^C enrichment in analyzed amino acids of untreated control cells and DCA treated cells. Results are shown for 9580 (**a**) and 10,158 cells (**b**). DCA treatment of 9580 and 10,158 cancer cell lines led to a significant decrease in ^13^C-enrichment in alanine (Ala), aspartate (Asp), glutamate (Glu), glycine (Gly), proline (Pro) and serine (ser). * *p* < 0.05, ** *p* < 0.01 *** *p* < 0.0001 compared to controls. Mass fragments of silylated amino acids used for ^13^C-determination are indicated.

In 9580 cancer cells, the incubation with DCA showed a significant decrease in overall ^13^C-incorporation into the amino acids alanine (4.1% treated vs. 9.8% controls), serine (2.1% vs. 3.4%), glutamate (2.6% vs. 6.7%), aspartate (1.8% vs. 4.0%), proline (0.3% vs. 1.2%) and glycine (2.0% vs. 2.3%; Figure 2, red arrows; Figure 3a). In the 10,158 cancer cells, DCA treatment measurement showed a significant decrease in overall ^13^C-incorporation into the amino acids alanine (1.8% treated vs. 5.6% in controls), serine (1.7% vs. 3.8%), glutamate (1.4% vs. 3.9%), aspartate (0.7% vs. 2.1%), proline (0.3% vs. 1.3%) and glycine (1.1% vs. 2.7%; Figure 3b).

#### 2.3.1. Isotopologue Profiles of Amino Acids from 9580 Cells

**Figure 4:**
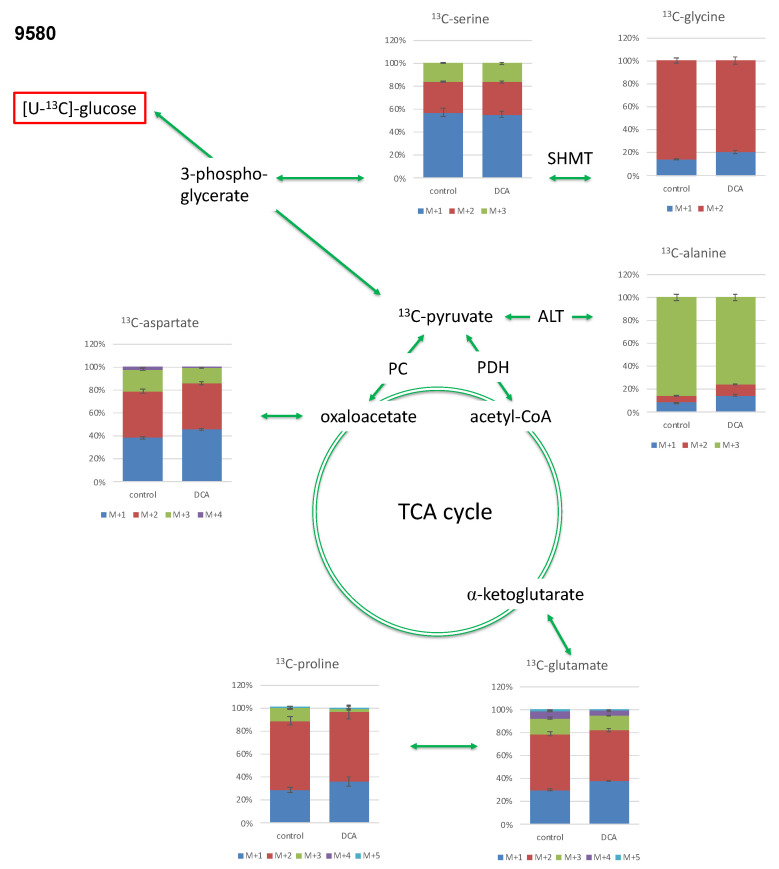
^13^C isotopologue profiles of serine, glycine, alanine, glutamate, proline and aspartate following incubation of 9580 cells with DCA compared to untreated controls. Relative fractions of isotopologues (M + 1–M + 5) in the overall ^13^C-labelled amino acids are indicated by different colors. ALT: alanine aminotransferase; PC: pyruvate carboxylase; PDH: pyruvate dehydrogenase; SHMT: serine hydroxymethyltransferase; *N* = 3.

In the 9580 cell line, the most abundant isotopologue of alanine was the [U-^13^C_3_]-species, which contained three ^13^C-atoms (out of three, this is denoted M + 3. Compared to the unlabelled species that carries three ^12^C-atoms, the ^13^C-isotopologue is characterized by an increase in three mass units due to three ^13^C-atoms (Figure 2 and Figure 4, Table 1)). The M + 2 isotopologues as the most abundant one were measured in glycine, aspartate, glutamate and proline. M + 1 was the predominant isotopologue in serine due to its formation from glycine via the serine hydroxymethyltransferase reaction (SHMT) using a ^13^C-labelled C_1_-source (Figure 4). The relative amount of the M + 3 isotopologue in alanine was significantly reduced in 9580 DCA-treated cells compared to untreated controls, whereas the relative fractions of the M + 1 and M + 2 species were significantly increased, especially in the case of alanine, aspartate, and glutamate. The isotopologue composition in serine and glycine was barely affected by the DCA exposure (Figure 4).

#### 2.3.2. Isotopologue Profiles of Amino Acids from 10,158 Cells

**Figure 5:**
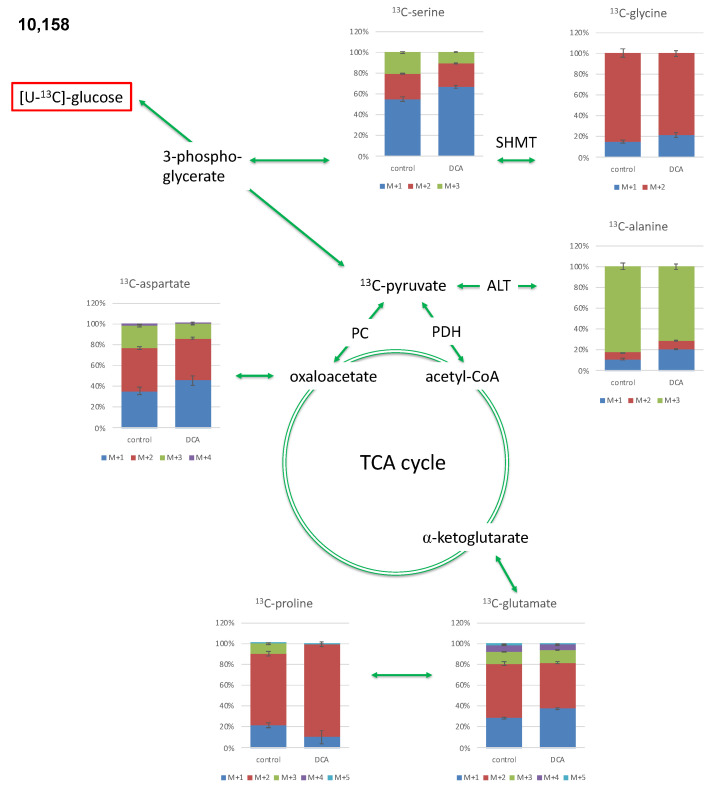
^13^C isotopologue profiles of serine, glycine, alanine, glutamate, proline and aspartate following incubation of 10,158 cells with DCA compared to untreated controls. Relative fractions of isotopologues (M + 1–M + 5) in the overall ^13^C-labelled amino acids are indicated by different colors. ALT: alanine aminotransferase; PC: pyruvate carboxylase; PDH: pyruvate dehydrogenase; SHMT: serine hydroxymethyltransferase; *N* = 3.

In the 10,158 cell line, the isotopologue profiles and the impacts upon these patterns due to DCA exposure resembled the ones from the 9580 cells (Figure 5 and Table 1).

The effects in Ala were slightly more pronounced in the 10,158 cells.

## 3. Discussion

One of the most striking hallmarks of cancer is that cancer cell proliferation is based on a high glycolytic turnover as well as abundant lactate production, which was first described by Otto Warburg [29]. By reprogramming their metabolism, cancer cells sustain their energy production through glycolysis despite the presence of oxygen, which is regarded to be a more efficient way of energy maintenance [30]. It was proposed that the salts of DCA result in shifting the metabolism of cancer cells from glycolysis to oxidative phosphorylation by indirect activation of pyruvate dehydrogenase (PDH) [31,32]. This would result in elevated flux into the citrate cycle (TCA). Yet, the effects of DCA on cellular, molecular metabolism have not been investigated in metabolic detail using these pancreatic cancer cells.

In our experimental setting, two pancreatic cancer cell lines were incubated in a medium containing a mixture of 5 mM of unlabelled glucose, 5 mM of [U-^13^C_6_]glucose and 0.1 mM glutamine. By using this specific cell medium composition with low glutamine concentration, we aimed to stimulate cellular glycolytic flux. A low amount of glutamine in the medium was necessary to maintain cellular proliferation, as a deficit of glutamine can lead to apoptosis in human fibroblasts, which is not the case for glucose [33]. For this experimental study, we took advantage of the well-established GC/MS-based ^13^C-analysis of amino acids to investigate metabolic pathways and carbon fluxes [23]. The analysis of the overall ^13^C-content in selected amino acids, as well as isotopologue patterns of both untreated pancreatic cancer cell lines, demonstrated the fluxes from the ^13^C-labelled-glucose into the characteristic precursors of the respective amino acids. These are reflective of the central metabolic intermediates of glycolysis or the citrate (TCA) cycle (see also Figure 2). Using this approach, direct effects of DCA upon PDH activation can therefore be monitored through the detailed analysis of the labelling data in aspartate or glutamate/proline derived from the TCA-cycle intermediates oxaloacetate or alpha-ketoglutarate, respectively. However, the chosen growth conditions also gleaned effects upon glycolytic flux and earlier metabolic targets of DCA.

According to the literature, DCA has effects on the depolarization of the mitochondria of cancer cells but does not influence the mitochondria of noncancerous cells [18,34]. At a concentration of 0.5 mM and incubation for 48 h, DCA significantly depolarized the mitochondria of A549, MO59K and MCF-7 cancer cells but did not affect healthy SAEC cells [34]. Additionally, in human ovarian cancer cells, A2780 and their cisplatin-resistant clones A2780/DDP, DCA treatment with concentrations of 40–80 mM upregulated apoptosis [35]. In HEK293, AGS and MKN45 gastric cancer cell concentrations of 0–50 mM of DCA were used, and cells were incubated for 24 h [36]. In the nontumorous cell line HEK293, the least change in glucose uptake and lactate production was observed compared to the other cell lines at a concentration between 20 mM and 50 mM [36]. Choosing a concentration of 75 mM DCA is comparably high compared to other studies with a range between 1 mM and 5 mM in Neuroblastoma cells [37], and in another neuroblastoma cell line, SK-N-SH and the breast cancer cell line SkBr3, with different concentrations ranging between 0.1 mM and 30 mM [38]. However, in another study with ovarian cancer cells, concentrations of 40 mM to 80 mM were used, based on the observation that cells A2780/DDP, that have a higher rate of glycolysis, are less sensitive to DCA, requiring 80 mM of DCA compared to A2780 cells, in which 40 mM of DCA already significantly inhibited cell viability and induced apoptosis [35]. In A2780/DDP cells, the mitochondrial OXPHOS level was low compared to A2780 cells [35]. With respect to normal cells, DCA has the potential to induce apoptosis in cancer stem cells but not in normal cells in mice treated with DCA (0.075 g/L, drinking water) or etoposide [39]. However, in human colorectal cancer cells, DCA did not result in significant alterations of metabolism or cell growth [40]. Furthermore, it was shown that DCA resulted only in apoptosis above concentrations of more than 25 mM IC50 after 48 h of treatment [41]. To conclude, the effects depend on cancer types, and treatment response can currently not be predicted.

Interestingly, in both PI3K mutated cell lines, glycolysis was a predominant pathway. Here, the ^13^C-patterns of alanine were especially interesting because its direct precursor is pyruvate, which is the product of glycolysis. The metabolism of alanine to pyruvate is driven by alanine aminotransferase (ALT), a process in which the isotopologue distribution from pyruvate is derived. Pyruvate synthesis is primarily realized via glycolysis originating from [U-^13^C_6_]glucose; hence, the high frequency of the detected [U-^13^C_3_]-isotopologue (M + 3) in alanine becomes evident (see Figure 2). The lower amounts of isotopologues M + 2 and M + 1 are mainly provided through catalytic action from malate through the malic acid enzyme or gluconeogenesis from oxaloacetate with some minor anabolic metabolism. Additionally, pyruvate can be provided through phosphoenolpyruvate, and the latter is metabolized by phosphoenolpyruvate carboxykinase (PEPCK). As a consequence of oxaloacetate metabolism, alanine will have the respective M + 2 and M + 1 labeling pattern. This expected isotopologue distribution was indeed measured in aspartate (amination product of oxaloacetate), which sustains a partial metabolic flux upstream via gluconeogenesis albeit abundancy of glucose in the cell culture medium. In both cell lines, glycolysis is the predominant metabolic driver, a conclusion that is based on an observed ratio of the relative ^13^C distribution in alanine of M + 3 and M + 2/M + 1 (>80%). Moreover, fluxes via the malic acid enzyme or gluconeogenesis were low (see Table 1 and Figure 2).

In amino acids derived from TCA-intermediates, the labeling profiles clearly reflected the expected patterns via [1,2-^13^C_2_]acetyl-CoA (derived from [U-^13^C_3_]pyruvate in the PDH-catalyzed reaction). Following the reaction in the TCA cycle, this results in ^13^C_2_-labelled alpha-ketoglutarate and oxaloacetate and the predominant M + 2 isotopologues in glutamate/proline and aspartate, respectively (Figure 2).

**Figure 6:**
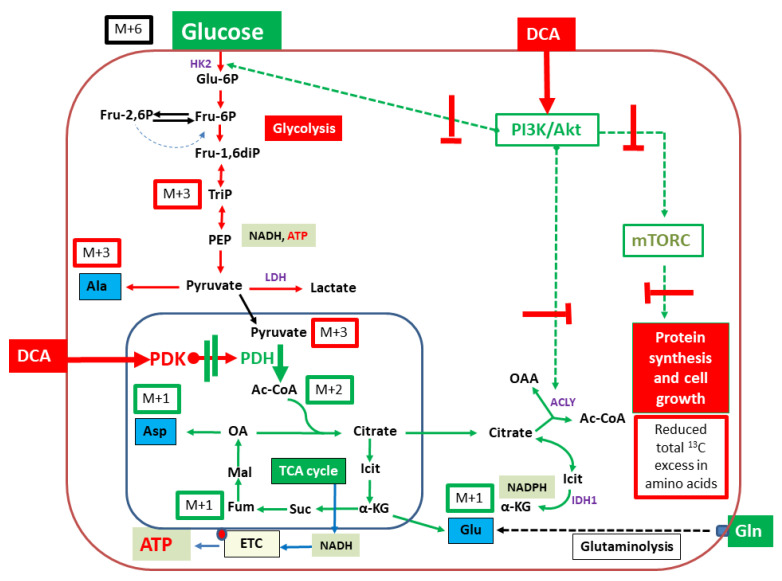
Model for DCA-induced effects upon the core metabolism in 9580 and 10,158 cancer cells. Green arrows and symbols indicate increased metabolic activities and stimulatory effects. Red arrows and symbols indicate reduced metabolic activities and inhibitory effects. Notably, glucose utilization via glycolysis could be reduced due to the deactivation of PI3K/Akt. Flux via the TCA cycle and oxidative phosphorylation could be increased by PDH activation due to inhibition and deactivation of PDK. When starting from [U-^13^C_6_]glucose (M + 6) as a carbon substrate, effects on the relative fractions of ^13^C-isotopologues in key metabolites and amino acids (in blue boxes) are indicated in red when reduced by DCA or in green when increased by DCA. M + 3, M + 2 and M + 1 indicate molecules carrying three, two or one ^13^C-atoms, respectively. PDH, pyruvate dehydrogenase; PDK, pyruvate dehydrogenase kinase, ETC, electron transfer chain; HK2, hexokinase 2; ACLY, ATP-citrate lyase; IDH1, isocitrate dehydrogenase 1.

The impacts of the treatment with DCA upon the above described ^13^C-labelling patterns were similar for both studied cell lines. Both cell lines demonstrated a DCA-treatment induced decrease in glycolytic metabolism upon incubation with DCA with a statistically significant lower total excess in ^13^C-enrichment in alanine, aspartate and glutamate (Figure 3). It is notable that the relative fraction of the M + 3 isotopologues in the labeled alanine was especially reduced in line with a significant down-regulation of glycolysis converting M + 6 glucose into M + 3 alanine (cf. Figure 6). It is also striking that the relative fractions of M + 1 isotopologue in aspartate, glutamate and proline were increased due to the DCA treatment. These species can be explained by multiple repetitions in the TCA cycle of M + 2 labeled oxaloacetate with unlabeled acetyl-CoA, which leads to the formation of ^13^C_1_-intermediates and the M + 1 isotopologues in Glu/Pro and Asp [23]. On this basis, the expected inhibitory effect of DCA on PDK resulting in activation of PDH and increased TCA cycling can indeed be observed, further confirming the validity of the experimental approach (Figure 6). However, more striking is the strong reduction in ^13^C-excess in all amino acids under study. We propose that this effect is due to inhibition of the PI3K/Akt/mTORC axis by DCA. As shown in Figure 6, DCA could affect one or more kinases involved in the PI3K and Akt regulatory network, thus affecting mTORC and resulting in reduced protein/amino acid biosynthesis. Moreover, uptake and glucose utilization could also be directly reduced by the decreased activation via PI3K/Akt (Figure 6). The observation of inhibitory effects of DCA upon the signal cascade via PI3K/Akt was previously proposed in LoVo cells [42]. Similar effects were also observed in pulmonary arterial smooth muscle cells with DCA-mediated inhibition of the Akt/GSK-3β pathway [43]. In retinoblastoma, a combination of DCA treatment led to the inhibition of the PI3K/Akt pathway and reduction in PDK1 protein levels [44].

The M + 1 species in glutamate, proline and aspartate are generated when unlabeled acetyl-CoA is transferred to M + 2 oxaloacetate. Hence, an increase in M + 1 upon DCA treatment indicates that acetyl-CoA sources other than glucose play an increased role. Such acetyl-CoA sources could be increased beta-oxidation [45] or glucogenic amino acids from increased autophagy [42]. The hypothesis of increased autophagy is corroborated by the previously mentioned increased M + 1 and M + 2 isotopologues in serine, glycine and alanine upon DCA treatment, which indicate increased flux through malic enzyme or gluconeogenesis. However, an increased activation of PDH would also lead to an increase in the metabolism of unlabeled pyruvate to acetyl-CoA, which in return results in a condensation of M + 2 oxaloacetate with unlabeled acetyl-CoA.

Using our approach, a comparison of the labelling data results of both untreated pancreatic cancer cells points out that glucose uptake and metabolic flux through glycolysis and the TCA cycle are more effective in 9580 cells. This observation can be derived from the higher overall ^13^C-content and comparison of ^13^C-labels in 9580 to 10,158 cells in aspartate, glutamate and alanine (see Figure 3). However, in the 9580 cells compared to the 10,158 cells, the ^13^C-content in the amino acids serine and glycine was lower. This is potentially due to a higher metabolism of external (unlabelled) serine by the 10,158 cells. The employment of this metabolic pathway would possibly need NAD^+^. The production of serine via glucose involves two oxidation reactions, which consume NAD^+^ and result in the formation of NADH [46]. For maximal proliferation, it was shown exemplarily in A549, MDA-MB-468 and MDA-MB-231 cells that sources of exogenous serine are needed [46]. Using our approach showed that in both cell lines, DCA acts already upstream of PDH. This can be derived from the decreased flux via 3-phosphoglycerate as deducible by low ^13^C-enrichment profiles of serine and glycine in 9580 and 10,158 cells. Interestingly, direct effects of DCA are assumed for PDK inhibition. This could indicate that additional subcellular therapeutic effects of DCA might contribute to this effect in vitro.

With reference to proline, ^13^C-enrichment was higher in 10,158 cells than in 9580, which possibly indicates an ameliorated metabolism from arginine via ornithine. Our working hypothesis is that this might be required to mobilize nitrogen or to sustain energy from the TCA cycle. However, no direct link to treatment response is available.

Interestingly, both pancreatic cancer cell lines showed a highly similar relative abundance of the different ^13^C isotopologues. Using this approach with [U-^13^C_6_]glucose, deeper insights into cellular metabolism become feasible and allow us to extend our knowledge beyond the initial step of glycolysis as approachable with diagnostics using [^18^F]FDG-PET. [^18^F]FDG is confined intracellularly after phosphorylation. However, [^18^F]FDG PET has limitations, as approximately 30% of cancers cannot be detected by [^18^F]FDG-PET [47]. This is the case because either a subset of tumors do not demonstrate sufficient glucose uptake or employ other metabolic pathways to supply their energetic cellular demands [47].

In terms of understanding metabolism, further limitations can be observed using [^18^F]FDG-PET, as subsequent steps of the glycolytic pathway are not directly accessible. To overcome this, ^13^C-enriched glucose or intermediates of glycolysis such as ^13^C labeled pyruvic acid are promising candidates. ^13^C is naturally decreased in plentitude (ca. 1%) [48] and therefore enabling targeted investigation of cellular metabolism with magnetic resonance spectroscopy and magnetic resonance imaging (MRI). MRI experiments at thermal equilibrium require time-consuming measurements so that a reasonable signal-to-noise ratio (SNR) can be obtained. With the introduction of dynamic nuclear hyperpolarization, a gain of SNR by >10,000-fold was achieved so that in vitro and in vivo categorization of metabolism-related processes was possible [49]. One of the most frequently used substances in vitro and in vivo is ^13^C-labelled pyruvic acid, which is useful in cancer cell characterization through the measurement of pyruvate to lactate metabolism [50,51,52,53]. The hyperpolarization principle was also successfully applied to ^13^C-enriched glucose [54]. However, limitations exist with pre-deuterated hyperpolarized ^13^C-enriched glucose, such as the short T1 time of 8–10 s, from which it is a challenge to follow more than one step of glycolysis with magnetic resonance spectroscopy at a full resolution [55]. Within this short time interval, all necessary preparations have to be fulfilled (e.g., dissolution of the mixture and application). Recently, chemical shift imaging (CSI) experiments with a special focus on improving the post-processing procedure together with MRI were introduced as an alternative method [56]. A 30-fold increase of SNR could be achieved using this technique [56]. In order to identify possible interesting metabolic substrates exploitable for such a “metabolic” approach, our aim was to decipher the metabolic pathways of glycolytic intermediates using GC-MS in a first step using this pre-established protocol. With GC-MS using [U-^13^C_6_]glucose a precise quantification of cell metabolism can be realized without having to address the additional challenges of fast decay of hyperpolarized signals. In light of changes in glycolytic intermediates in both pancreatic cancer cell lines, future “focused” studies with hyperpolarized ^13^C glucose or non hyperpolarized MRS using CSI become reasonable.

To the authors’ knowledge, this is the first study that investigated the effects of DCA on the core cellular metabolism in pancreatic cancer cells using ^13^C-glucose labelling monitored by GC-MS. As a key finding, we could show that DCA already acts upstream of PDH, albeit at high concentrations. Given that treatment of pancreatic cancer is the still a major clinical challenge, further investigations on cellular level at high metabolic resolution might help to understand therapeutic related alterations. Thus, our results underscore the importance of a better understanding of cellular metabolism with the aim of potentially identifying new targets for combination treatments to enhance treatment success.

## 4. Materials and Methods

### 4.1. Cell Lines

For the in vitro experiments, the murine pancreatic cancer cell lines 10,158 and 9580 were used. Cells were cultivated in a humidified atmosphere at 37 °C containing 5% CO. Cell culture media consisted of DMEM (Biochrom AG, Berlin, Germany), plus 10% FCS, plus 1% Penicillin/Streptomycin. Both cell lines had mutated PI3K. The genotype of the 10,158 is Ptf1a-Cre;LSL-PI3KCAH1047R/+, the genotype of the 9580 is p48-Cre;LSL-PIK3CAH1047R/H1047R. Cell lines were characterized and gifted by Dieter Saur and Christian Veltkamp.

### 4.2. WST-1 Cell Viability/Proliferation Assay

In this study, sodium dichloroacetate (Sigma Aldrich, Taufkirchen, Germany) was used for the treatment of the cells. Cells were either left untreated (controls) or treated with the following concentrations of DCA for ca. 24 h: 1 mM, 10 mM, 25 mM, 50 mM, 75 mM, 100 mM. For quantification of effects of DCA treatment, 2.5 × 10^4^ were seeded in triplicate into 96 well plates (Greiner Bio One, Frickenhausen, Germany) 24 h before treatment. After the addition of 10 μL (per 100 μL medium) of a liquid reagent containing tetrazolium salt (WST-1 reagent, Roche Diagnostics, Penzberg, Germany), the metabolic activity of both cell lines (controls and cell treated with DCA) was measured. Changes in the formazan dye development were quantified 60 min after the addition of the WST-1 reagent in an ELISA Reader (Bio-Tek, Winooski, VT, USA). The reaction of the reagent is indicative of cellular metabolic activity. For ease of comparison, the results of controls were set to 100%. Therefore, results below 100% represent decreased viability/proliferation, and vice versa, those above 100% indicate increased viability/proliferation.

### 4.3. Treatment of Cells with DCA and Incubation with [U-^13^C_6_]Glucose

Cells were seeded in 175 cm^2^ culture flasks (approximately 5 × 10^6^ cells per flask) and adhered overnight. On the following day, cells (approximately 1 × 10^7^ cells per flask) were incubated with 75 mM of DCA for ca. 18 h and [U-^13^C_6_]glucose. Controls were treated accordingly, however with the difference that an equal volume of PBS was added and resuspended instead of the DCA solution. The [U-^13^C_6_]glucose culture medium contained unlabelled D-glucose (5 mM), [U-^13^C_6_]D-glucose (5 mM; 99.9% ^13^C-content, Sigma-Aldrich, Taufkirchen, Germany), glutamine (0.1 mM), glucose-free DMEM (Biochrom AG, Berlin, Germany) and 10% FCS. The cells were incubated in the incubator with 37 °C and 5% CO_2_. Following the defined incubation time, cells were harvested, the suspension was washed in PBS three times, and after centrifugation and removal of the supernatants, the cellular pellets were frozen at −80 °C until further use. Experiments were carried out as triplicates.

### 4.4. Isotopologue Profiling of Cell Lines Exposed to DCA

To analyze treatment associated metabolic alterations in 9580 and 10,158 cells upon DCA treatment, mock-treated (controls) and DCA-treated cells frozen at −80 °C were freeze-dried for 24 h (Martin Christ Gefriertrocknungsanlagen GmbH, Osterode, Germany). First, preparation of the amino acids consisted of the addition of 1 mL of 6 M HCl to 10 mg of freeze-dried cells (approximately 1 × 10^7^) and resuspended. Secondly, the reaction mixture was incubated for 24 h at 105 °C in a sealed tube. Thirdly, the cell hydrolysate was dried at 70 °C in a heat block applying a constant stream of N_2_ gas on the following day. To dissolve the residue, 200 μL of 50% acetic acid were added to the sample, and the mixture was sonicated for 1 min. Fourth, purification of amino acids was realized with a self-made column, which is composed of a 1 mL pipette tip filled with glass wool and the cation-exchange resin Dowex^®^ 50W-X8 (hydrogen form, 200–400 mesh, Alfa Aesar: L13922, Thermo Fisher Scientific, Waltham, MA, USA). Before applying the sample, the column was prepared by washing it once with 1 mL 70% methanol and thereafter twice with 1 mL H_2_O. The self-made column was washed twice with 1 mL H_2_O after the addition of the sample. One milliliter of 4 M NH_3_ solution was added in order to elute the amino acids. Fifth, a 200 μL-fraction of the 1 mL eluate was transferred to a glass vial. Sixth, the eluate was dried at 70 °C using N_2_ as described above. The remaining 800 µL of the eluate was stored at −20 °C in order to allow replicate analyses. Seventh, amino acids were converted into volatile silyl-derivatives. These derivates were subjected to GC/MS-analysis, as described earlier [57,58]. In detail, a suspension of 50 μL of water-free acetonitrile (ACN) and 50 μL of N-methyl-N-tert-butyldimethylsilyltrifluoroacetamide (MTBSTFA) containing 1% TBDMS chloride was created. These samples were incubated in a sealed GC-MS vial for 30 min at 70 °C. For analysis, these prepared samples were transferred to GS-MS microvials. The detection of selected amino acids was realized using a GC-MS instrument (GC 2010, GCMS-QP 2010, auto injector AOC-20i, Shimadzu, Munich, Germany).

### 4.5. Data Analysis

The evaluation of the GC/MS results was performed with the associated software “GCMSsolution” (Shimadzu, Duisburg, Germany). The calculation of the total excess and isotopologue distribution was performed in Excel (Microsoft GmbH, München, Germany) with custom scripts. The statistical analysis was realized with GraphPad Prism Version 5 (GraphPad Software, Inc., San Diego, CA, USA). For comparison, *t*-Tests were used, followed by Welch’s test. If *p* was <0.05, statistical significance was assumed.

## Figures and Tables

**Table 1:** Enrichment profiles and percentage of ^13^C-enrichment in six selected amino acids in controls and after treatment with DCA in 9580 and 10,158 cancer cell lines. * *p* < 0.05, ** *p* < 0.01 *** *p* < 0.0001 compared to controls. Experiments were carried out as triplicates.

	9580		10,158	
	Control	DCA	Control	DCA
Alanine				
M + 1	0.90% ± 0.03%	0.70% ± 0.02% **	0.68% ± 0.07%	0.45% ± 0.01% *
M + 2	0.65% ± 0.02%	0.44% ± 0.02% ***	0.40% ± 0.03%	0.18% ± 0.01% **
M + 3	9.02% ± 0.31%	3.60% ± 0.13% **	5.10% ± 0.20%	1.53% ± 0.06% **
Aspartate				
M + 1	3.28% ± 0.11%	1.99% ± 0.05% *	1.54% ± 0.16%	0.80% ± 0.08% *
M + 2	3.47% ± 0.13%	1.76% ± 0.06% **	1.83% ± 0.06%	0.71% ± 0.02% **
M + 3	1.63% ± 0.06%	0.59% ± 0.03% ***	0.93% ± 0.05%	0.24% ± 0.03% ***
M + 4	0.18% ± 0.01%	0.03% ± 0.00% **	0.07% ± 0.01%	0.00% ± 0.00% **
Glutamate				
M + 1	5.03% ± 0.14%	2.67% ± 0.05% **	2.76% ± 0.09%	1.38% ± 0.03% **
M + 2	8.12% ± 0.30%	3.14% ± 0.10% **	5.09% ± 0.15%	1.62% ± 0.04% ***
M + 3	2.29% ± 0.11%	0.84% ± 0.03% **	1.15% ± 0.05%	0.45% ± 0.03% **
M + 4	1.02% ± 0.07%	0.32% ± 0.01% **	0.61% ± 0.03%	0.18% ± 0.01% **
M + 5	0.24% ± 0.02%	0.06% ± 0.00% **	0.13% ± 0.00%	0.04% ± 0.00% ***
Glycine				
M + 1	0.35% ± 0.02%	0.47% ± 0.03% *	0.44% ± 0.04%	0.27% ± 0.03% **
M + 2	2.08% ± 0.06%	1.77% ± 0.07% *	2.49% ± 0.12%	0.97% ± 0.04% **
Proline				
M + 1	0.95% ± 0.07%	0.33% ± 0.04% ***	0.73% ± 0.08%	0.07% ± 0.04% **
M + 2	2.01% ± 0.12%	0.56% ± 0.05% **	2.31% ± 0.07%	0.57% ± 0.02% ***
M + 3	0.37% ± 0.04%	0.02% ± 0.02% **	0.33% ± 0.02%	0.00% ± 0.00% **
M + 4	0.00% ± 0.00%	0.00% ± 0.00%	0.00% ± 0.00%	0.00% ± 0.00%
M + 5	0.00% ± 0.00%	0.00% ± 0.00%	0.00% ± 0.00%	0.00% ± 0.00%
Serine				
M + 1	3.67% ± 0.23%	2.13% ± 0.10% **	3.80% ± 0.15%	2.34% ± 0.04% **
M + 2	1.75% ± 0.04%	1.09% ± 0.04% **	1.68% ± 0.05%	0.80% ± 0.02% **
M + 3	1.02% ± 0.03%	0.63% ± 0.04% **	1.41% ± 0.08%	0.38% ± 0.02% **

## Data Availability

All of the reported data is included in the manuscript.

## References

[1] Siegel R., Naishadham D., Jemal A. (2013). Cancer statistics, 2013. CA A Cancer J. Clin..

[2] Eser S., Schnieke A., Schneider G., Saur D. (2014). Oncogenic KRAS signalling in pancreatic cancer. Br. J. Cancer.

[3] Aichler M., Seiler C., Tost M., Siveke J., Mazur P.K., Da Silva-Buttkus P., Bartsch D.K., Langer P., Chiblak S., Dürr A. (2011). Origin of pancreatic ductal adenocarcinoma from atypical flat lesions: A comparative study in transgenic mice and human tissues. J. Pathol..

[4] Morris J.P., Wang S.C., Hebrok M. (2010). KRAS, Hedgehog, Wnt and the twisted developmental biology of pancreatic ductal adenocarcinoma. Nat. Rev. Cancer.

[5] Kanda M., Matthaei H., Wu J., Hong S., Yu J., Borges M., Hruban R.H., Maitra A., Kinzler K., Vogelstein B. (2012). Presence of Somatic Mutations in Most Early-Stage Pancreatic Intraepithelial Neoplasia. Gastroenterology.

[6] Collins M.A., Brisset J.-C., Zhang Y., Bednar F., Pierre J., Heist K.A., Galbán C.J., Galbán S., Di Magliano M.P. (2012). Metastatic Pancreatic Cancer Is Dependent on Oncogenic Kras in Mice. PLoS ONE.

[7] Collisson E.A., Sadanandam A., Olson P., Gibb W.J., Truitt M., Gu S., Cooc J., Weinkle J., Kim G.E., Jakkula L. (2011). Subtypes of pancreatic ductal adenocarcinoma and their differing responses to therapy. Nat. Med..

[8] Berndt N., Hamilton A.D., Sebti S.M. (2011). Targeting protein prenylation for cancer therapy. Nat. Rev. Cancer.

[9] Castellano E., Downward J. (2011). RAS Interaction with PI3K: More Than Just Another Effector Pathway. Genes Cancer.

[10] Pylayeva-Gupta Y., Grabocka E., Bar-Sagi D. (2011). RAS oncogenes: Weaving a tumorigenic web. Nat. Rev. Cancer.

[11] Britten C.D. (2013). PI3K and MEK inhibitor combinations: Examining the evidence in selected tumor types. Cancer Chemother. Pharmacol..

[12] Michelakis E.D., Sutendra G., Dromparis P., Webster L., Haromy A., Niven E., Maguire C., Gammer T.L., Mackey J.R., Fulton D. (2010). Metabolic Modulation of Glioblastoma with Dichloroacetate. Sci. Transl. Med..

[13] Liang Y., Zhu D., Zhu L., Hou Y., Hou L., Huang X., Li L., Wang Y., Li L., Zou H. (2019). Dichloroacetate Overcomes Oxaliplatin Chemoresistance in Colorectal Cancer through the miR-543/PTEN/Akt/mTOR Pathway. J. Cancer.

[14] Kolesnik D., Pyaskovskaya O., Yakshibaeva Y., Solyanik G. (2019). Time-dependent cytotoxicity of dichloroacetate and metformin against Lewis lung carcinoma. Exp. Oncol..

[15] Verma A., Lam Y.-M., Leung Y.-C., Hu X., Chen X., Cheung E., Tam K.Y. (2019). Combined use of arginase and dichloroacetate exhibits anti-proliferative effects in triple negative breast cancer cells. J. Pharm. Pharmacol..

[16] Li B., Li X., Xiong H., Zhou P., Ni Z., Yang T., Zhang Y., Zeng Y., He J., Yang F. (2017). Inhibition of COX2 enhances the chemosensitivity of dichloroacetate in cervical cancer cells. Oncotarget.

[17] Abildgaard C., Dahl C., Abdul-Al A., Christensen A., Guldberg P. (2017). Inhibition of retinoic acid receptor beta signaling confers glycolytic dependence and sensitization to dichloroacetate in melanoma cells. Oncotarget.

[18] Michelakis E.D., Webster L., Mackey J.R. (2008). Dichloroacetate (DCA) as a potential metabolic-targeting therapy for cancer. Br. J. Cancer.

[19] Stacpoole P.W. (1989). The pharmacology of dichloroacetate. Metabolism.

[20] James M.O., Yan Z., Cornett R., Jayanti V.M., Henderson G.N., Davydova N., Katovich M.J., Pollock B., Stacpoole P.W. (1998). Pharmacokinetics and metabolism of [14C]dichloroacetate in male Sprague-Dawley rats. Identification of glycine conjugates, including hippurate, as urinary metabolites of dichloroacetate. Drug Metab. Dispos..

[21] Yan Z., Henderson G.N., James M.O., Stacpoole P.W. (1997). Determination of dichloroacetate and its metabolites in human plasma by gas chromatography–mass spectrometry. J. Chromatogr. B Biomed. Sci. Appl..

[22] Shroads A.L., Coats B.S., Langaee T., Shuster J.J., Stacpoole P.W. (2015). Chloral hydrate, through biotransformation to dichloroacetate, inhibits maleylacetoacetate isomerase and tyrosine catabolism in humans. Drug Metab. Pers. Ther..

[23] Gkiouli M., Biechl P., Eisenreich W., Otto A. (2019). Otto Diverse Roads Taken by 13C-Glucose-Derived Metabolites in Breast Cancer Cells Exposed to Limiting Glucose and Glutamine Conditions. Cells.

[24] Feuerecker B., Biechl P., Seidl C., Bruchertseifer F., Morgenstern A., Schwaiger M., Eisenreich W. (2021). Diverse metabolic response of cancer cells treated with a 213Bi-anti-EGFR-immunoconjugate. Sci. Rep..

[25] Otto A.M. (2020). Metabolic Constants and Plasticity of Cancer Cells in a Limiting Glucose and Glutamine Microenvironment—A Pyruvate Perspective. Front. Oncol..

[26] Davidson S.M., Papagiannakopoulos T., Olenchock B.A., Heyman J.E., Keibler M.A., Luengo A., Bauer M.R., Jha A.K., O’Brien J.P., Pierce K.A. (2016). Environment Impacts the Metabolic Dependencies of Ras-Driven Non-Small Cell Lung Cancer. Cell Metab..

[27] Fan T.W.M., Lane A.N., Higashi R.M., A Farag M., Gao H., Bousamra M., Miller D.M. (2009). Altered regulation of metabolic pathways in human lung cancer discerned by 13C stable isotope-resolved metabolomics (SIRM). Mol. Cancer.

[28] Hensley C.T., Faubert B., Yuan Q., Lev-Cohain N., Jin E., Kim J., Jiang L., Ko B., Skelton R., Loudat L. (2016). Metabolic Heterogeneity in Human Lung Tumors. Cell.

[29] Warburg O.H., Dickens F., Kaiser-Wilhelm-Institut for Biology (1930). The Metabolism of Tumours; Investigations from the Kaiser Wilhelm Institute for Biology, Berlin-Dahlem.

[30] Hanahan D., Weinberg R.A. (2011). Hallmarks of Cancer: The Next Generation. Cell.

[31] Tataranni T., Piccoli C. (2019). Dichloroacetate (DCA) and Cancer: An Overview towards Clinical Applications. Oxidative Med. Cell. Longev..

[32] Kankotia S., Stacpoole P.W. (2014). Dichloroacetate and cancer: New home for an orphan drug?. Biochim. Biophys. Acta Bioenerg..

[33] Yuneva M., Zamboni N., Oefner P., Sachidanandam R., Lazebnik Y. (2007). Deficiency in glutamine but not glucose induces MYC-dependent apoptosis in human cells. J. Cell Biol..

[34] Bonnet S., Archer S.L., Allalunis-Turner J., Haromy A., Beaulieu C., Thompson R., Lee C.T., Lopaschuk G.D., Puttagunta L., Bonnet S. (2007). A Mitochondria-K+ Channel Axis Is Suppressed in Cancer and Its Normalization Promotes Apoptosis and Inhibits Cancer Growth. Cancer Cell.

[35] Zhou L., Liu L., Chai W., Zhao T., Jin X., Guo X., Han L., Yuan C. (2019). Dichloroacetic acid upregulates apoptosis of ovarian cancer cells by regulating mitochondrial function. OncoTargets Ther..

[36] Hur H., Xuan Y., Kim Y.B., Lee G., Shim W., Yun J., Ham I.-H., Han S.-U. (2012). Expression of pyruvate dehydrogenase kinase-1 in gastric cancer as a potential therapeutic target. Int. J. Oncol..

[37] Feuerecker B., Seidl C., Pirsig S., Bruchelt G., Senekowitsch-Schmidtke R. (2015). DCA promotes progression of neuroblastoma tumors in nude mice. Am. J. Cancer Res..

[38] Feuerecker B., Pirsig S., Seidl C., Aichler M., Feuchtinger A., Bruchelt G., Senekowitsch-Schmidtke R. (2012). Lipoic acid inhibits cell proliferation of tumor cells in vitro and in vivo. Cancer Biol. Ther..

[39] Morfouace M., Lalier L., Bahut M., Bonnamain V., Naveilhan P., Guette C., Oliver L., Gueguen N., Reynier P., Vallette F.M. (2012). Comparison of Spheroids Formed by Rat Glioma Stem Cells and Neural Stem Cells Reveals Differences in Glucose Metabolism and Promising Therapeutic Applications*. J. Biol. Chem..

[40] Ho N., Coomber B.L. (2015). Pyruvate dehydrogenase kinase expression and metabolic changes following dichloroacetate exposure in anoxic human colorectal cancer cells. Exp. Cell Res..

[41] Stockwin L.H., Yu S.X., Borgel S., Hancock C., Wolfe T.L., Phillips L.R., Hollingshead M.G., Newton D.L. (2010). Sodium dichloroacetate selectively targets cells with defects in the mitochondrial ETC. Int. J. Cancer.

[42] Gong F., Peng X., Sang Y., Qiu M., Luo C., He Z., Zhao X., Tong A. (2013). Dichloroacetate induces protective autophagy in LoVo cells: Involvement of cathepsin D/thioredoxin-like protein 1 and Akt-mTOR-mediated signaling. Cell Death Dis..

[43] Li B., Zhu Y., Sun Q., Yu C., Chen L., Tian Y., Yan J. (2018). Reversal of the Warburg effect with DCA in PDGFtreated human PASMC is potentiated by pyruvate dehydrogenase kinase1 inhibition mediated through blocking Akt/GSK3beta signalling. Int. J. Mol. Med..

[44] Sradhanjali S., Tripathy D., Rath S., Mittal R., Reddy M.M. (2017). Overexpression of pyruvate dehydrogenase kinase 1 in retinoblastoma: A potential therapeutic opportunity for targeting vitreous seeds and hypoxic regions. PLoS ONE.

[45] Misbin R.I. (1979). Effects of dichloroacetate on lipid metabolism in isolated rat liver cells. Diabetes.

[46] Diehl F.F., Lewis C.A., Fiske B.P., Heiden M.G.V. (2019). Cellular redox state constrains serine synthesis and nucleotide production to impact cell proliferation. Nat. Metab..

[47] Jones R.G., Thompson C.B. (2009). Tumor suppressors and cell metabolism: A recipe for cancer growth. Genes Dev..

[48] Eakin R., Morgan L., Gregg C., Matwiyoff N. (1972). Carbon-13 nuclear magnetic resonance spectroscopy of living cells and their metabolism of a specifically labeled13C substrate. FEBS Lett..

[49] Serrao E.M., Brindle K.M. (2017). Dynamic nuclear polarisation: The future of imaging in oncology?. Porto Biomed. J..

[50] Feuerecker B., Durst M., Michalik M., Schneider G., Saur D., Menzel M., Schwaiger M., Schilling F. (2017). Hyperpolarized 13C Diffusion MRS of Co-Polarized Pyruvate and Fumarate to Measure Lactate Export and Necrosis. J. Cancer.

[51] Feuerecker B., Michalik M., Hundshammer C., Schwaiger M., Bruchertseifer F., Morgenstern A., Seidl C. (2019). Assessment of 213Bi-anti-EGFR MAb treatment efficacy in malignant cancer cells with [1-13C]pyruvate and [18F]FDG. Sci. Rep..

[52] Bliemsrieder E., Kaissis G., Grashei M., Topping G., Altomonte J., Hundshammer C., Lohöfer F., Heid I., Keim D., Gebrekidan S. (2021). Hyperpolarized 13C pyruvate magnetic resonance spectroscopy for in vivo metabolic phenotyping of rat HCC. Sci. Rep..

[53] Hundshammer C., Braeuer M., Müller C.A., Hansen A.E., Schillmaier M., Düwel S., Feuerecker B., Glaser S.J., Haase A., Weichert W. (2018). Simultaneous characterization of tumor cellularity and the Warburg effect with PET, MRI and hyperpolarized 13C-MRSI. Theranostics.

[54] Mishkovsky M., Anderson B., Karlsson M., Lerche M.H., Sherry A.D., Gruetter R., Kovacs Z., Comment A. (2017). Measuring glucose cerebral metabolism in the healthy mouse using hyperpolarized 13C magnetic resonance. Sci. Rep..

[55] Singh J., Suh E.H., Sharma G., Khemtong C., Sherry A.D., Kovacs Z. (2019). Probing carbohydrate metabolism using hyperpolarized 13 C-labeled molecules. NMR Biomed..

[56] Brender J.R., Kishimoto S., Merkle H., Reed G., Hurd R.E., Chen A.P., Ardenkjaer-Larsen J.H., Munasinghe J., Saito K., Seki T. (2019). Dynamic Imaging of Glucose and Lactate Metabolism by (13)C-MRS without Hyperpolarization. Sci Rep..

[57] Eylert E., Herrmann V., Jules M., Gillmaier N., Lautner M., Buchrieser C., Eisenreich W., Heuner K. (2010). Isotopologue profiling of Legionella pneumophila: Role of serine and glucose as carbon substrates. J. Biol. Chem..

[58] Eylert E., Schar J., Mertins S., Stoll R., Bacher A., Goebel W., Eisenreich W. (2008). Carbon metabolism of Listeria monocytogenes growing inside macrophages. Mol. Microbiol..

